# Draft Genome Sequence of *Stenotrophomonas bentonitica* BII-R7^T^, a Selenite-Reducing Bacterium Isolated from Spanish Bentonites

**DOI:** 10.1128/genomeA.00719-17

**Published:** 2017-08-03

**Authors:** Iván Sánchez-Castro, Mohammed Bakkali, Mohamed L. Merroun

**Affiliations:** a Departamento de Microbiología, Universidad de Granada, Granada, Spain; b Departamento de Genética, Universidad de Granada, Granada, Spain

## Abstract

The Gram-negative bacterium *Stenotrophomonas bentonitica* BII-R7^T^ was isolated from bentonite formations. Like other species within the genus *Stenotrophomonas*, strain BII-R7^T^ possesses high tolerance to numerous heavy metals, suggesting potential for bioremediation purposes. The draft genome sequence reported here comprises 4.37 Mb with a G+C content of 66.5% and 3,796 predicted protein-coding sequences.

## GENOME ANNOUNCEMENT

*Stenotrophomonas bentonitica* BII-R7^T^ (= LMG 29893^T^ = CECT 9180^T^ = DSM 103927^T^) is a recently described Gram-negative bacterial strain that was isolated from bentonite formations located in southern Spain (1). The genus *Stenotrophomonas* has hitherto comprised 14 established species, isolated from a large variety of environments (2–14), that are resistant to certain antibiotics and metals (15). In this sense, *S. bentonitica* has shown high uranium (1) and selenium (M. A. Ruiz-Fresneda, unpublished data) tolerance due to different interaction mechanisms, suggesting potential applicability for bioremediation purposes. Indeed, the biotechnological use of *Stenotrophomonas* spp. has already been proposed (16–21). Research on all but one of the 14 known *Stenotrophomonas* spp., *S. tumulicola* (13), counts on freely available genome sequences of the corresponding species (22). Here, we report the draft genome sequence of *S. bentonitica* strain BII-R7^T^.

After cultivation on LB medium, genomic DNA of *S. bentonitica* BII-R7^T^ was extracted as described by Martín-Platero et al. (23). A genomic library with an insert size of 350 bp was sequenced using the Illumina HiSeq 2000 platform at Macrogen, Inc. (Seoul, Republic of Korea).

A total of 53,608,108 paired-end 101-bp reads were obtained (>1,000 × coverage). The quality of the reads was assessed using FastQC (24), and the Q20 and Q30 indices were 95.34% and 87.69%, respectively. Multiple *de novo* genome assemblies were performed using ABySS version 1.5.1 (25) with *k*-mer sizes between 19 and 95. The assemblies were merged, filtered, and further assembled into scaffolds using TransABySS (26) and GS *de novo* assembler software (Roche). We obtained 191 scaffolds with an *N*_50_ of 35,432 and an *L*_50_ of 38. The mean size of these scaffolds was 22,890 bp with the largest comprising 187,875 bp and the smallest comprising 2,262 bp. The size of the entire sequence was 4,371,992 bp with a 66.5% G+C content, which are values in accordance with described *Stenotrophomonas* spp.

Gene prediction and annotation were performed using the Rapid Annotations using Subsystems Technology server (27) and the Prokaryotic Genome Annotation Pipeline (28). The genomic features of *S. bentonitica* BII-R7^T^ included a total of 3,786 coding sequences (CDSs), 1 complete rRNA cluster, 44 tRNAs, 4 ncRNAs, and 158 pseudogenes. The coding sequences were classified into 431 subsystems, the most abundant of which were for the metabolism of amino acid derivatives (*n* = 352 CDSs); carbohydrates (*n* = 214); protein metabolism (*n* = 205); metabolism of cofactors, vitamins, prosthetic groups, and pigments (*n* = 204); membrane transport (*n* = 160); and RNA metabolism (*n* = 147). Additionally, 115 of these coding sequences were related to stress responses, such as osmotic and oxidative stress, cold and heat shock stress, or uptake of selenate and selenite. Genes related to degradation or resistance to a variety of toxic compounds (e.g., ethidium bromide) and heavy metals (e.g., cobalt, zinc, cadmium, tellurium, copper, arsenic, or mercury) were also identified in the present draft genome. Moreover, the draft genome contains specific enzymes, such as alkaline and acid phosphatases or glutathione reductases, which could, respectively, be involved in the high levels of tolerance that *S. bentonitica* BII-R7^T^ (1, 17) has to uranium and selenium.

### Accession number(s).

This whole-genome shotgun project has been deposited at GenBank/ENA/DDBJ under the accession number MKCZ00000000. The version described in this paper is the first version, MKCZ01000000.
